# Validity of instruments to measure physical activity may be questionable due to a lack of conceptual frameworks: a systematic review

**DOI:** 10.1186/1477-7525-9-86

**Published:** 2011-10-03

**Authors:** Elena Gimeno-Santos, Anja Frei, Fabienne Dobbels, Katja Rüdell, Milo A Puhan, Judith Garcia-Aymerich

**Affiliations:** 1 Centre for Research in Environmental Epidemiology (CREAL), Barcelona, Spain; 2 Hospital del Mar Research Institute (IMIM), Barcelona, Spain; 3 CIBER Epidemiología y Salud Pública (CIBERESP), Barcelona, Spain; 4 Horten Centre for Patient-oriented Research, University Hospital of Zurich, Switzerland; 5 Institute of General Practice and Health Services Research, University Hospital of Zurich, Switzerland; 6 Centre for Health Services and Nursing Research, Katholieke Universiteit Leuven, Leuven, Belgium; 7 Patient Reported Outcomes Centre of Excellence, Market Access, Primary Care Business Unit, Pfizer Ltd, Sandwich, Kent, UK; 8 Department of Epidemiology, Johns Hopkins Bloomberg School of Public Health, Johns Hopkins University, Baltimore (MD), USA; 9 Department of Experimental and Health Sciences, Universitat Pompeu Fabra (UPF), Barcelona, Spain

**Keywords:** Chronic heart disease, chronic respiratory disease, conceptual framework, elderly, patient reported outcomes, physical activity, questionnaire, systematic review

## Abstract

**Background:**

Guidance documents for the development and validation of patient-reported outcomes (PROs) advise the use of conceptual frameworks, which outline the structure of the concept that a PRO aims to measure. It is unknown whether currently available PROs are based on conceptual frameworks. This study, which was limited to a specific case, had the following aims: (i) to identify conceptual frameworks of physical activity in chronic respiratory patients or similar populations (chronic heart disease patients or the elderly) and (ii) to assess whether the development and validation of PROs to measure physical activity in these populations were based on a conceptual framework of physical activity.

**Methods:**

Two systematic reviews were conducted through searches of the Medline, Embase, PsycINFO, and Cinahl databases prior to January 2010.

**Results:**

In the first review, only 2 out of 581 references pertaining to physical activity in the defined populations provided a conceptual framework of physical activity in COPD patients. In the second review, out of 103 studies developing PROs to measure physical activity or related constructs, none were based on a conceptual framework of physical activity.

**Conclusions:**

These findings raise concerns about how the large body of evidence from studies that use physical activity PRO instruments should be evaluated by health care providers, guideline developers, and regulatory agencies.

## Background

Patient-reported outcome (PRO) instruments have always been an important tool in epidemiological and clinical research. Recently, interest in these instruments has increased with their use as outcome measures in randomized trials of pharmacological and non-pharmacological interventions. Regulatory agencies, namely the United States Food and Drug Administration (FDA) and the European Medicines Agency (EMA), have developed guidance documents concerning the appropriate development, validation, and use of PRO instruments in clinical trials [1,2]. Particular emphasis has been placed on their validity, that is, the ability of a PRO to measure the concept that it is intended to measure. To this end, the use of conceptual frameworks is advised [3-6]. The conceptual framework explicitly defines the concepts measured by the instrument in a diagram that represents the relationships between the main concept (e.g., health-related quality of life), the domains (e.g., symptoms), the sub-domains (e.g., dyspnea), and the items measured as well as the scores obtained from a PRO instrument [2,7]. An absent or inadequate conceptual framework is likely to lead to inadequate development and validation of a PRO [3-6], which in turn, may create confusion about what is actually being measured [7].

The proportion of PROs for which a conceptual framework formed the basis for the development and validation process is currently unknown. For regulatory agencies and stakeholders such as patients and physicians, it is only possible to understand the meaning of the effects of health care interventions on PROs if the underlying concepts to be measured are clearly outlined. Because PROs represent a very broad group of outcomes, it would be overly ambitious to assess all types of PROs that have been developed. Therefore, we focused on PROs that capture aspects of physical activity as the main concept and chronic respiratory diseases as the main study subjects. Physical activity is a key concept in public health because reduced physical activity is a well-known risk factor for many chronic diseases and disorders [8], and sedentary lifestyles are common around the world [9]. Despite the importance of physical activity, it is challenging to define what physical activity actually means and how to capture the important aspects of physical activity. Thus, a conceptual framework of physical activity is particularly important for instruments that intend to measure this parameter. We focused on chronic respiratory diseases for two reasons: they are a leading cause of morbidity and mortality worldwide [9], and respiratory health is not included in most physical activity recommendations, despite the epidemiological and clinical evidence that regular physical activity may reduce the incidence and improve the prognosis of chronic respiratory diseases [10-12]. The current gap between research and public health needs may be partly due to the absence of a universally accepted definition of physical activity in studies of patients with chronic respiratory (and similar) diseases.

The aims of this study were (i) to identify available conceptual frameworks of physical activity in chronic respiratory patients or similar populations, and (ii) to determine whether the development and validation of currently used PRO instruments to measure physical activity in these populations were based on a conceptual framework of physical activity.

## Methods

This study was part of the European Union-funded PROactive project (http://www.proactivecopd.com), which aims to develop, validate, and apply patient-reported outcome instruments to capture the dimensions of physical activity in daily life relevant to patients with chronic obstructive pulmonary disease (COPD). The PROactive consortium is multidisciplinary and includes academic partners, patient organizations, and pharmaceutical companies.

We utilized standard systematic review methodology following the handbooks of the Centre for Reviews and Dissemination [13] and the Cochrane Collaboration [14]. The manuscript follows the PRISMA [15] statement for reporting of systematic reviews and meta-analyses. All methods were specified in advance, documented in a protocol, and approved by the PROactive consortium. This manuscript includes data from two systematic reviews performed as part of the PROactive project. First, a systematic literature search, detailed below, was conducted to identify conceptual frameworks of physical activity. Second, we performed a secondary analysis from another systematic review of the PROactive consortium [unpublished observation] that identified existing PRO instruments for measuring physical activity.

### Systematic review of conceptual frameworks of physical activity

#### Eligibility criteria

The following eligibility criteria were applied:

1. Type of studies: Any type of discussion article (e.g., seminar articles, viewpoints, unsystematic reviews or similar articles) that proposed and discussed a conceptual framework of physical activity, as defined by the FDA ("the conceptual framework explicitly defines the concepts measured by the instrument in a diagram that presents a description of the relationships between items, domain (subconcepts), and concepts measured and the scores produced by a PRO instrument"). We considered articles that were specifically focused on physical activity and excluded research articles in which only some parts of the introduction or discussion sections addressed physical activity. No language, publication date, or publication status restrictions were imposed.

2. Type of population: Elderly people (≥60 years of age) or subjects over 40 years of age with any of the following conditions: chronic respiratory disease (COPD, asthma or interstitial lung disease), symptomatic coronary heart disease, or congestive heart failure.

3. Type of information: Descriptions of what constitutes physical activity (the concept of the conceptual framework) and how it may be measured by domains, sub-domains, and, ultimately, items. We did not consider articles that described a concept of physical activity but lacked specifying domains (because they did not fulfill the conditions of a conceptual framework, which requires the specification of domains). Physical activity was defined as "any bodily movement produced by skeletal muscles which results in energy expenditure" [16]. This definition of physical activity includes activities such as activities of daily living, sports, and activities for personal fulfillment.

#### Information sources and search

We performed searches of the following electronic databases: Medline, Embase, CINAHL, and PsycINFO. We used the following search terms: chronic obstructive lung disease, interstitial lung disease, asthma, emphysema, coronary disease, heart failure, elderly, physical activity, motor activity, activity of daily living, physical inactivity, theoretical framework, conceptual framework, patient reported, patient self-reported, patient perception, control group, cross-over studies, meta-analysis, epidemiological studies, cohort studies, cross-sectional studies, and seroepidemiologic studies [see Additional file 1]. All publications prior to January 2010 (the time of the most recent search) were included. Additionally, because we expected that some documents on conceptual frameworks may not be published in the public domain and that electronic searches may miss relevant articles because of inconsistent indexing of articles in databases, we also performed manual searches of (i) all references listed in retrieved full-text articles and (ii) the first 50 references (sorted by link ranking) from PubMed's "Related Articles" search filter of retrieved full-text articles. We also contacted external scientists on this topic to identify further articles.

#### Management of references

The bibliographic details of all retrieved articles were stored in a RefWorks-COS file; RefWorks is a software program that is particularly helpful for organizing title and abstract screening by authors from remote sites. We removed duplicate records resulting from the database searches. The source of the identified articles (database, hand-search, expert contacts) was recorded in a "user-defined field" in each RefWorks-COS file. Additional user-defined fields were assigned to individual reviewers, who recorded their decisions for inclusion and exclusion.

#### Study selection

Two independent reviewers assessed the title and abstract of each identified citation. The decisions of the reviewers (order or reject) were recorded in the RefWorks-COS file and compared. Any disagreements were resolved by consensus, with close attention to the previously defined inclusion/exclusion criteria. Two independent reviewers evaluated the retrieved full text of all potentially eligible articles and made a decision on inclusion or exclusion according to the predefined selection criteria. Any disagreements were resolved by consensus, with close attention to the inclusion/exclusion criteria. In the case of a persistent disagreement, a third reviewer decided upon inclusion or exclusion. All studies that did not fulfill all of the predefined criteria were excluded, and their bibliographic details were listed with the specific reason for exclusion.

#### Data collection process

We developed a data extraction Microsoft^® ^Office Excel sheet. Because the number of included studies was very small, a random pilot test was not feasible. To overcome this limitation and to avoid losing relevant information, two reviewers independently tested the form, which was refined prior to the final extraction process. The final version of the data extraction form was used by three independent reviewers to screen the full text of the included studies. Any disagreements were resolved by consensus, with close attention to the data extraction criteria.

#### Data extraction

The following information was extracted from each included study: (i) bibliographic details such as author, journal, year of publication, and language and (ii) details about the characteristics of conceptual frameworks and definitions of domains.

#### Quality of studies

Given the type of studies considered (no empirical data with estimates), the assessment of the quality of the studies is not applicable.

#### Summary measures

We summarized the conceptual frameworks. In addition, we drew a graph for each framework that included the concept being measured (level 1), its domains (level 2), their sub-domains, if applicable (level 3), and their items (level 4). We contacted the authors of the included studies, who confirmed that our graphs and descriptions appropriately represented the conceptual frameworks they proposed.

### Systematic review of PRO instruments for measuring physical activity

We used data from a recent systematic review to determine which conceptual frameworks were used to support the development and validation of current PRO instruments for measuring physical activity. The detailed methods of that review are described elsewhere [unpublished observation].

#### Study selection

Two independent reviewers evaluated the retrieved full text of the 103 articles presenting PROs to assess physical activity previously included in the original review [unpublished observation] and excluded those not based on the type of population defined above (elderly people (≥60 years of age) or subjects over 40 years of age with any of the following conditions: chronic respiratory disease (COPD, asthma or interstitial lung disease), symptomatic coronary heart disease, congestive heart failure), and those not based on a conceptual framework. Any disagreements were resolved by consensus, with close attention to the inclusion/exclusion criteria. In the case of a persistent disagreement, a third reviewer decided upon inclusion or exclusion. All studies that did not fulfill all of the predefined criteria were excluded, and their bibliographic details were listed with the specific reason for exclusion.

#### Data extraction

We developed a data extraction Microsoft^® ^Office Excel sheet, pilot tested it with a random sample of ten studies, and refined it accordingly. Two independent reviewers extracted the data, and any disagreements were resolved by consensus, with close attention to the data extraction criteria. The following information was extracted from each included study: (i) bibliographic details such as author, journal, year of publication, and language; (ii) whether the instrument was based on a conceptual framework; (iii) whether the conceptual framework was defined prior to statistical analysis, defined after statistical analysis, or refined after statistical analysis; (iv) the main concept and its definition; and (v) the domains and their definitions.

#### Summary measures

We summarized the results in a table and detailed all data extracted.

## Results

### Systematic review of conceptual frameworks of physical activity

**Figure 1:**
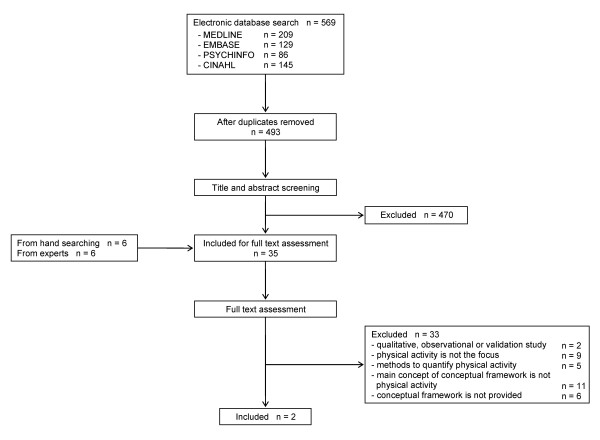
**Flow diagram of process of systematic literature search**.

A total of 569 references were identified from electronic database searches [Figure 1]. After deleting duplicates, 493 references remained. From these, 470 were excluded after screening based on the titles and abstracts. Therefore, 23 papers from the database searches, in addition to 6 additional papers obtained by hand-search and 6 papers provided by the experts, were included for full-text assessment. Of these papers, we excluded 33 articles for not focusing on physical activity (n = 9) or not providing a conceptual framework of physical activity (n = 11). Finally, 2 papers provided a conceptual framework that included the main concept, domains, sub-domains and potential items and thus were included in the review. Both papers provided conceptual frameworks of physical activity in COPD patients. We did not identify any conceptual framework for physical activity in other chronic respiratory diseases, symptomatic coronary heart disease, congestive heart failure, or elderly people.

**Figure 2:**
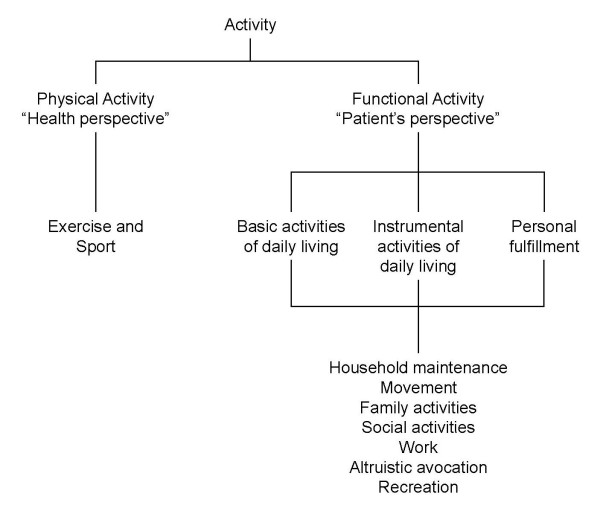
**Conceptual framework proposed by Leidy (COPD, 2007)**.

The article by Leidy [Figure 2] provided a conceptual framework based on qualitative research and expert opinion [17]. The author suggested that measuring activity should be broader than simply quantifying the amount of physical activity (e.g., as time spent on moderate or strenuous physical activity), which only reflects the perspective of health care professionals who want to increase people's physical activity levels to improve health outcomes ("health promotion"). The author argued that "functional activity", which has been identified in qualitative research as important to patients, should also be considered and should include activities of daily living (basic and instrumental) and personal fulfillment. We interpreted "activity" to be the main concept of the conceptual framework (level 1) and "physical activity-health perspective" and "functional activity-patient's perspective" to be the domains (level 2), as confirmed by the author The author provided examples of sub-domains and items for the "functional activity" domain derived from a previous paper on qualitative research in COPD patients [18].

**Figure 3:**
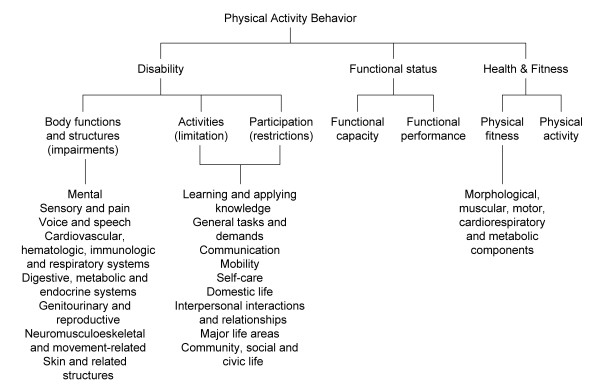
**Conceptual framework proposed by Larson (COPD, 2007)**.

Larson provided a conceptual framework embedded in a rehabilitation context [19]. The author proposed a framework based on the International Classification of Functioning, Disability and Health (ICF) framework [20], the functional status framework [21], and the President's Fitness Council model [22]. We interpreted "physical activity behavior" to be the main concept, with "disability", "functional status", and "health & fitness" as the domains (level 2), again confirmed by the author. See Figure 3 for details on the sub-domains and items.

### Systematic review of PRO instruments for measuring physical activity

From 103 studies of PRO instruments measuring physical activity, the dimensions of physical activity or related constructs, 45 studies (44%) did not satisfy the population inclusion criteria, and 36 studies (35%) were not based on a conceptual framework. None of the questionnaires with physical activity as the main concept was based on conceptual frameworks of physical activity derived from previous research or expert knowledge. Thus, 22 instruments (21%) based on a conceptual framework were included for data extraction. Their details are displayed in Table 1. None of these 22 instruments included physical activity as the main concept of the PRO conceptual framework, and only 7 (32%) considered physical activity as a domain. Most of the studies defined a conceptual framework prior to statistical analysis of the psychometric properties of the instrument. Only one defined its conceptual framework after the analysis, and three papers refined the domains after a factor analysis [see Table 1].

**Table 1:** Data Extraction Based on Physical Activity Questionnaires (n = 22)

Author, year	Population	Instrument	Article includes a conceptual framework		**Main concept**^† ^**and definition**	**Domains**^†^
			*A priori*	*A posteriori*		
Arbuckle, 1994 [27]	Elderly	Activities Checklist	x	-	Activity level Definition not reported	- Intellectual activity - Social and **physical activity**
Avlund, 1996 [28]	Elderly	Questionnaire of Functional Ability	x	-	Functional ability Definition not reported	**-Physical Activities of Daily Living **(PADL) **- Instrumental Activities of Daily Living **(IADL)
Carone, 1999 [29]	COPD, kiphoscoliosis	Maugery Foundation Respiratory Failure item set (MRF-28)	-	x (domains)	Health impairment Definition not reported	**- Daily activities** - Cognitive function - Invalidity
Dunderdale, 2008 [30]	Chronic Heart Failure	Chronic Heart Failure Assessment tool (CHAT)	x	x (domains)	Health related quality of life Definition not reported	*A priori:* - Physical - Emotional - Self-perception - Relationships - Symptoms - Lifestyle - Cognitive aspects *A posteriori:* - Symptoms - Activity levels - Psychosocial aspects - Emotions
Eakman, 2007 [31]	Elderly	Meaningful Activity Participation Assessment (MAPA)	x	-	Meaningful Activity Participation Definition not reported	- Mental health - Purpose in life - Physical health

**Author, year**	**Population**	**Instrument**	**Article includes a conceptual framework**		**Main concept**^† ^**and definition**	**Domains**^†^
			***A priori***	***A posteriori***		

Fillenbaum, 1981 [32]	Elderly	OARS Multidimensional Functional Assessment Questionnaire	x	-	Personal functioning Definition not reported	- Social - Economic - Mental healthy - Physical health - Self capacity
Kempen, 1990 [33]	Elderly	Hierarchial Polychotomous ADL-IADL Scale	x	-	Functioning in daily life Definition not reported	**- ADL** **- IADL**
Laureau, 1994 [34]	COPD	Pulmonary Functional Status and Dyspnea Questionnaire (PFSDQ)	x	-	Dyspnea 'The sensation of uncomfortable breathing' [...] 'in patients with chronic obstructive pulmonary disease (COPD), is the primary symptom limiting activities of daily living'	- Dyspnea components - Functional abilities
Laureau, 1998 [35]	COPD	Modified version of the Pulmonary Functional Status and Dyspnea Questionnaire (PFSDQ-M)	x	-	Activity levels 'Activity levels based on the patient's self-report of his or her perception in performing 79 activities' Dyspnea 'Patient's experience with dyspnea, followed by ratings of the intensity of shortness of breath experienced with the performance of the same 79 activities evaluated in the activity component'	- Self-care - Mobility - Eating - Home management - Social activities - Recreational activities

**Author, year**	**Population**	**Instrument**	**Article includes a conceptual framework**		**Main concept**^† ^**and definition**	**Domains**^†^
			***A priori***	***A posteriori***		

Lee, 1998 [36]	Various pulmonary disease	University of Cincinnati Dyspnea Questionnaire (UCDQ)	x	-	Dyspnea 'The subjective perception of difficult or laboured breathing. Difficult breathing in patients with pulmonary disease has been cited as the single most important factor limiting their ability to function on a day-to-day basis'	- Speech - Physical - Combination
Leidy, 1999 [37]	COPD	Functional Performance Inventory (FPI)	x	-	Functional Status 'A multidimensional concept characterizing one's ability to provide for the necessities of life-those activities people do in the normal course of their lives meet basic needs, fulfil usual roles, and maintain their health and well-being'	- Functional capacity - Functional performance - Functional reserve - Capacity utilization
Letrait, 1996 [38]	Astma	Asthma Impact Record (AIR) index	x	x	Asthma-related health status Definition not reported	After interviews' patients (*a priori*): **- Physical activity **(mobility) - Symptoms - Psychological, - Social and - Acceptability of the disease and treatment After analysis (*a posteriori*): **- Physical activities** - Physical symptoms - Psychological and - Social dimension

**Author, year**	**Population**	**Instrument**	**Article includes a conceptual framework**		**Main concept**^† ^**and definition**	**Domains**^†^
			***A priori***	***A posteriori***		

Maille, 1997 [39]	Asthma, Chronic Bronchitis and Emphysema	Quality of Life Respiratory Illness Questionnaire (QOL-RIQ)	x	x (domains)	Disease-specific Quality of Life Definition not reported	*A priori:* - Physical and Functional status - Psychological status - Social functioning *A posteriori:* - Breathing problems - Physical problems - Emotions - General activities - Situations triggering or enhancing breathing problems **- Daily and domestic activities** - Social activities, relationship and sexuality
Migliore, 2006 [40]	COPD	Dyspnea Management Questionnaire (DMQ)	x	-	Dyspnea 'The perception and experienced of laboured, uncomfortable breathing derived from interactions among multiple physiological, psychological, social and environmental factors, and may induce secondary physiological and behavioural responses'	- Dyspnea and related anxiety with activities - Appraisal of dyspnea coping skills

**Author, year**	**Population**	**Instrument**	**Article includes a conceptual framework**		**Main concept**^† ^**and definition**	**Domains**^†^
			***A priori***	***A posteriori***		

Morimoto, 2003 [41]	COPD	Chronic Obstructive Pulmonary Disease Activity Rating Scale (CARS)	x	-	Life-related activities 'The dimension that deals with all aspects of human life in accordance with the International Classification of Functioning and Disability'	- Self care - Domestic, - Outdoor and - Social interaction
Morris, 1989 [42]	Elderly	IOWA Self-Assessment Inventory (ISAI)	x	-	Functional characteristics Definition not reported	- Social resources - Economic resources - Mental health - Physical health - **ADL** - Cognitive status
Schultz-Larsen, 1992 [43]	Elderly	Questionnaire of Functional Ability	x	-	Functional ability Definition not reported	- Tiredness - Reduced speed
Tu, 1997 [44]	COPD	The Seattle Obstructive Lung Disease Questionnaire (SOLQ)	x	-	Health-Related Quality of Life Definition not reported	- Physical function - Emotional function - Coping skills - Treatment satisfaction
Van der Molen, 2003 [45]	COPD	Clinical COPD Questionnaire (CCQ)	x	-	Health Related Quality of Life 'Functional effect of an illness and its consequent therapy upon a patient, as perceived by the patient'	- Functional status - Symptoms - Mental state

**Author, year**	**Population**	**Instrument**	**Article includes a conceptual framework**		**Main concept**^† ^**and definition**	**Domains**^†^
			***A priori***	***A posteriori***		

Wigal, 1991 [46]	COPD	COPD Self-Efficacy Scale (CSES)	x (main concept)	x (domains)	Self efficacy 'Personal convictions people have regarding whether or not they feel they can successfully execute particular behaviours in order to produce certain outcomes'	- Negative affect - Intense emotional arousal - Physical exertion - Weather/environment - Behavioural risk factors
Zaragoza, 2009 [47]	COPD, asthma	The Quality of Life Questionnaire for Patients With Chronic Respiratory Disease (CV-PERC)	x	-	Health-Related Quality of Life 'The subjective perception of how a disease and its treatment affect different aspects of a patient's everyday life'	- Physical functioning - Psychological functioning - Social functioning - Cognitive functioning - Sexual functioning - Perceived well-being and health - Work functioning
Zisberg, 2005 [48]	Elderly	Scale of Older Adults' Routine (SOAR)	x	-	Routine 'Is a concept pertaining to strategically designed behavioural patterns used to organize and coordinate activities along the axes of time, duration, social and physical contexts, sequence and order'	- Basic activities - Instrumental activities - Rest - Leisure activities - Social participation and work - Volunteering

^†^In bold when the terms as we defined as "physical activity" if they are included in the main concept or domains.

## Discussion

This review identified only 2 conceptual frameworks for physical activity in COPD patients, whereas no conceptual frameworks seem to exist for patients with chronic heart disease, or elderly people. Furthermore, none of the available instruments for measuring dimensions of physical activity or related constructs in these populations was based on a conceptual framework for physical activity. These results may reflect the incomplete understanding of what physical activity means in chronic respiratory disease patients and similar populations (i.e., chronic heart disease patients or the elderly).

A potential limitation of this review is that some conceptual frameworks for physical activity may have been missed, despite a rigorous database search followed by a comprehensive hand-search and communication with expert. The indexing of this type of publication is not standardized, thus creating challenges in identifying relevant publications. Another potential limitation is that the FDA guidance for the PRO measures was published in 2006, whereas most of the PRO instruments included in our reviews were developed prior to that date. However, as early as 1985, previous guidelines for developing questionnaires included the requirement of a conceptual framework [3-6], even if this was labeled differently. The strengths of our review are the inclusion of chronic respiratory disease patients, patients with chronic cardiac diseases and elderly people, and the use of the same population criteria and concept definitions within two reviews.

A challenge of this review was understanding the definitions and concepts presented in the articles included in the full-text assessment because of the inconsistent use of terminology and the overlap between domains, sub-domains, and items within each conceptual framework. To resolve this issue, the authors strictly applied the definition of a conceptual framework (a concept to be measured by domains, sub-domains, and, ultimately, items). It was apparent that most articles considered for full-text assessment did not focus on physical activity as the concept to be measured or did not present a conceptual framework. Similarly, identifying a conceptual framework from the manuscripts developing PRO instruments was complicated because most articles provided the main concept, but the identification of their conceptual framework was much more difficult. A general recommendation from our review is that manuscripts should maintain consistency in the labeling of main concepts and domains, and in their definitions. Additionally, a discrepancy may be observed between the number of conceptual frameworks identified in the first (n = 2) and second reviews (n = 22). It is important to emphasize that none of the conceptual frameworks identified in the second review had physical activity as the main concept; consequently, they cannot be considered to represent conceptual frameworks of physical activity.

The requirement that PRO instrument development should be based on a conceptual framework has been long established and acknowledged in several guides and standards, such as the Medical Outcomes Trust [4,5], Health Measurements Scales [6] and the American Psychological Association (APA) guidelines [3]. In particular, the *Standards for Educational and Psychological Testing *of the APA state that "the construct of interest for a particular test should be embedded in a conceptual framework, no matter how imperfect that framework may be" [3]. Our finding of a lack of conceptual frameworks for physical activity is in agreement with the lack of conceptual frameworks identified by O'Brien *et al. *for the comprehensive evaluation and treatment of people living with HIV [23]. Unfortunately, no other papers similar to this paper have been identified, which supports the view that there is little awareness and knowledge of this topic. Our second finding, that less than half of the PROs for physical activity or related constructs were based on a conceptual framework, is consistent with the finding of Pollard *et al. *that none of the PRO instruments for health outcome measures in osteoarthritis were based on a conceptual framework [24]. Of additional interest, we noted that only 3 questionnaires out of 22 refined their domains after the analysis, despite the need to develop PRO instruments through an iterative process, per FDA recommendations [25]. All things considered, our study suggests that there is limited knowledge of the complete process of developing and validating a PRO instrument.

It is debatable whether physical activity is a construct that requires a conceptual framework for its definition given the current trend in activity monitoring. The authors' opinion, based on the current state-of-the-art, is that physical activity is a multifaceted construct that goes well beyond the amount or frequency of physical activity. The concept of physical activity could also include, for example, the ability to perform activities of daily living and symptoms such as dyspnea or pain associated with physical activity, which are not captured by accelerometers or pedometers. Irrespective of the number of domains included in the construct of physical activity, it is critical that the use of questionnaires to measure it should be based on a previous conceptual framework.

The lack of conceptual frameworks for physical activity by individuals with chronic diseases such as COPD also has public health implications. In the meeting report entitled *Implementation of the World Health Organization (WHO) Strategy for Prevention and Control of Chronic Respiratory Diseases*, increasing physical activity levels was included in the recommendations for the development of policies in the area of chronic respiratory diseases [25]. The lack of a conceptual framework seems to preclude the success of potential interventions because there is no clear concept based upon which to intervene. One could question whether this lack of definition of physical activity is responsible, at least in part, for the lack of effectiveness of most physical activity interventions in chronic-disease patients at the population level [26]. A second potentially negative effect of the lack of conceptual frameworks is the difficulty of measuring the effectiveness of the interventions because instruments may not exactly measure what they claim to measure. Furthermore, using a PRO that is not based on a conceptual framework may lead to measurement error (information bias), which is a challenge to detect intervention effects. Therefore, we argue that (new) conceptual frameworks for physical activity for the elderly and for populations with chronic respiratory diseases and chronic heart diseases must be derived. These frameworks should precede the development of new PRO instruments for physical activity in these populations.

## Conclusion

We identified only 2 conceptual frameworks for physical activity in COPD. We found that none of the currently available PROs that aim to measure physical activity in chronic respiratory disease patients or similar populations (chronic heart disease patients or the elderly) are based on a conceptual framework with physical activity as the main concept. Our findings contrast sharply with the FDA's guidance on the development and validation of PROs, raising the question of how the large body of evidence from trials that use these instruments should be evaluated by health care providers, guideline developers, and regulatory bodies.

## List of Abbreviations

APA: American Psychological Association; COPD: Chronic Obstructive Pulmonary Disease; EMEA: European Medicines Agency; FDA: Food and Drug Administration; ICF: International Classification of Functioning, Disability and Health; PRO: Patient Reported Outcome; WHO: World Health Organization.

## Competing interests

The authors declare that they no have competing interests.

## Authors' contributions

MP and JGA led the systematic review. EGS, AF, FD, KR, MP and JGA developed the study protocol. AF and EGS coordinated the review. EGS and AF conducted the electronic database searches; EGS conducted the additional searches. AF coordinated the references in RefWorks. AF and MP (1st reviewers) and EGS and JGA (2nd reviewers) screened titles and abstracts. AF and MP (1st reviewers) and EGS and JGA (2nd reviewers) assessed full texts of the identified studies and extracted the relevant data. EGS, MP and JGA drafted the manuscript. All authors contributed to revising the manuscript and approved the final version. PROactive consortium approved the final version of the manuscript.

## Supplementary Material

Additional file 1 **Search strategy: MEDLINE, EMBASE, CINAHL and PSYCHINFO**. Outline of the search strategy used for electronic database searching. Click here for file
