# Supplementary material for: Validity of instruments to measure physical activity may be questionable due to a lack of conceptual frameworks: a systematic review
Source: Health Qual Life Outcomes. 2011 Oct 3;9:86. doi: 10.1186/1477-7525-9-86 (PMC3215640; doi:10.1186/1477-7525-9-86)
Supplement: Additional file 1 — Search strategy: MEDLINE, EMBASE, CINAHL and PSYCHINFO. Outline of the search strategy used for electronic database searching. [file 1477-7525-9-86-S1.PDF]

Search strategy: MEDLINE, EMBASE, CINAHL and PSYCHINFO

1. exp lung diseases, interstitial/ or exp lung diseases, obstructive/ or exp pulmonary emphysema/
2. (COPD or 'chronic lung disease' or 'chronic obstructive lung disease' or 'chronic bronchitis' or emphysema or asthma or Interstitial Lung Disease or 'Lung Disease, Interstitial' or 'Interstitial Lung Diseases' or 'Diffuse Parenchymal Lung Diseases').tw.
3. exp Coronary Disease/
4. exp Heart Failure/
5. ('Coronary Diseases' or 'Coronary Herat Disease' or 'Coronary Heart Diseases' or 'Cardiac Failure' or 'Myocardial Failure' or 'Left-Sided Heart Failure' or 'Right SIDEC Heart Failure' or 'Congestive Heart Failure' or 'Heart Decompensation').tw.
6. exp Aged/
7. (elderly or 'frail elderly' or aged).tw.
8. 6 or 7
9. asthma.mp. or exp Asthma/
10. 8 and 9
11. frail elderly.mp. or exp Frail Elderly/
12. exp *Aged/
13. exp *Aged/px [Psychology]
14. 12 not 13
15. 10 or 11 or 14
16. 1 or 2
17. 3 or 4 or 5
18. 15 or 16 or 17
19. exp Motor Activity/
20. exp "Activities of Daily Living"/
21. exp Exercise/ph [Physiology]
22. ((measur* or assess*) adj10 ("physical activit*" or 'Motor activit*' or 'activit* of daily living' or 'Chronic Limitation of Activit*' or 'Limitation of Activit*' or 'Daily Living Activit*' or "motor inactivit*" or 'physical inactivit*' or "functional activit*" or "functional performance")).tw.
23. or/19-22
24. 18 and 23
25. ((measur* or assess*) adj3 (activit* or performance or function)).tw.
26. exp "Outcome Assessment (Health Care)"/mt [Methods]
27. ((abilit* or disabilit*) adj3 (measur* or assess*)).tw.

28. ((outcome adj3 measurement) or (outcome adj3 assessment) or (theor* adj3 framework) or (concept* adj 3 framework) or (questionnaire adj3 design) or (assessment adj3 model) or ((basic or generic) adj3 concept)).tw.
29. or/25-28
30. 24 and 29
31. control groups/ or double-blind method/ or meta-analysis as topic/ or patient selection/ or random allocation/ or simple size/
32. control groups/ or cross-over studies/ or double-blind method/ or matched-pair analysis/ or meta-analysis as topic/ or random allocation/ or "reproducibility of results"/ or sample size/ or "sensitivity and specificity"/ or single-blind method/
33. epidemiologic studies/ or case-control studies/ or cohort studies/ or cross-sectional studies/ or seroepidemiologic studies/
34. or/31-33
35. 30 not 34
36. limit 35 to clinical trial, all
37. limit 36 to "all child (0 to 18 years)"
38. child*.ti.
39. 36 or 37 or 38
40. 35 not 39
41. (functional adj3 (capacit* or limitation* or abilit*)).ti.
42. (performance or activit*).ti.
43. (patient reported or patient-reported or patient centered or patient-centered or self-report* or questionnaire*).tw.
44. (patients adj3 (view or perception or experience)).tw.
45. or/41-44
46. 40 and 45
47. trial.ti.
48. 46 not 47
49. limit 48 to animals
50. (Subjective Measurement of Activity in Chronic Obstructive Pulmonary Disease or Measuring functional performance in patients with COPD).ti.
51. 48 and 50
